# Innovative Polymeric Hybrid Nanocomposites for Application in Photocatalysis

**DOI:** 10.3390/polym13081184

**Published:** 2021-04-07

**Authors:** Maria Cantarella, Giuliana Impellizzeri, Alessandro Di Mauro, Vittorio Privitera, Sabrina Carola Carroccio

**Affiliations:** 1 CNR-IMM, Via S. Sofia 64, 95123 Catania, Italy; maria.cantarella@ct.infn.it (M.C.); alessandro.dimauro@ct.infn.it (A.D.M.); 2 CNR-IMM, Z.I. VIII Strada 5, 95121 Catania, Italy; vittorio.privitera@cnr.it; 3 CNR-IPCB, Via Paolo Gaifami 18, 95126 Catania, Italy; sabrinacarola.carroccio@cnr.it

**Keywords:** photocatalysis, polymeric nanocomposites, hybrid materials, water treatment, nanomaterials, atomic layer deposition, adsorbent materials

## Abstract

The immobilization of inorganic nanomaterials on polymeric substrates has been drawing a lot of attention in recent years owing to the extraordinary properties of the as-obtained materials. The hybrid materials, indeed, combine the benefits of the plastic matter such as flexibility, low-cost, mechanical stability and high durability, with them deriving from their inorganic counterparts. In particular, if the inorganic fillers are nanostructured photocatalysts, the originated hybrid systems will be able to utilize the energy delivered by light, catalysing chemical reactions in a sustainable pathway. Most importantly, since the nanofillers can be ad-hoc anchored to the macromolecular structure, their release in the environment will be prevented, thus overcoming one of the main restrictions that impedes their applications on a large scale. In this review, several typologies of hybrid photocatalytic nanomaterials, obtained by using both organic and inorganic semiconductors and realized with different synthetic protocols, were reported and discussed. In the first part of the manuscript, nanocomposites realized by simply blending the TiO_2_ or ZnO nanomaterials in thermoplastic polymeric matrices are illustrated. Subsequently, the atomic layer deposition (ALD) technique is presented as an excellent method to formulate polymeric nanocomposites. Successively, some examples of polyporphyrins hybrid systems containing graphene, acting as photocatalysts under visible light irradiation, are discussed. Lastly, photocatalytic polymeric nanosponges, with extraordinary adsorption properties, are shown. All the described materials were deeply characterized and their photocatalytic abilities were evaluated by the degradation of several organic water pollutants such as dyes, phenol, pesticides, drugs, and personal care products. The antibacterial performance was also evaluated for selected systems. The relevance of the obtained results is widely overviewed, opening the route for the application of such multifunctional photocatalytic hybrid materials in wastewater remediation.

## 1. Introduction

Technological developments constantly require innovative products and solutions to be more and more competitive and sustainable in the market. In this context, nanomaterials (with at least one dimension smaller than 100 nm) play a key role in a wide range of sectors covering medicine [1], energy [2], the food industry [3], agriculture [4], and environmental science [5]. Indeed, they show superior performance if compared to the related bulk state, mainly associated with their high surface area and shapeable form that magnify peculiar properties, such us the chemical reactivity [6]. Although the nano-structuration offers outstanding physical and chemical properties that are today exploited in new material and engineering science, the release into a natural media and/or final disposal of such kinds of “atomic level objects” constitutes a debatable topic [7,8,9,10]. Consequently, irrespective of their application, the fate of nanomaterials should be carefully taken into account. As an example, the intriguing use of nanoclays in drug and gene delivery cannot still find a widespread clinical application due to the lack of information on their adverse effects on human health [11]. This deal is also particularly relevant in the case of wastewater treatment where the well-established photodegradation efficiency of TiO_2_ nanoparticles (NPs) in degrading organic pollutants cannot be proficiently employed due to time and energy-consuming filtration processes needed to ensure “safe” water effluents. The latter aspect constitutes the most important technological limitation for a future commercialization [12].

To make available the properties of nanomaterials, their coating and/or immobilization in a suitable organic support appears to be the key solution for real technological applications [13]. This strategy can brilliantly overcome several difficulties such as the necessary recovery of TiO_2_ NPs from treated water [12]. Among the various organic substrates, polymers cover a leading position, being the most promising materials for their great tunability in function of the final application. Flexibility, low-cost production, mechanical stability, and durability are features required for energy [14], packaging [15], and environmental applications [16], whereas in the cases of biomedical applications, biocompatibility is also desired, together with some specific stimuli responsive ability [17]. Since the assembling of such materials, which considerably differ in chemo-physical and morphological properties, is a difficult task to achieve, many efforts have been devoted to efficiently embed the nanomaterials into polymers, realizing the so-called polymeric hybrid nanocomposites [18,19,20,21].

The efforts of the scientific community in finding a good match among nanomaterials, polymeric support, and formulation method, have been mainly dedicated to the water remediation issues. This is not surprising since the major research challenge of our century is to guarantee a constant access to clean water, avoiding and/or removing contamination sources. In this view, the recent advancements in nanotechnology offer unprecedented and viable solutions for the realization of next-generation water treatment systems [22,23,24]. It is worth noticing that despite the increasing demand of clean water owing to the rapid growth of world population and industries, traditional treatment technologies are obsolete and are not adequate to manage and remove emerging pollutants such us drugs, pesticides, and personal care chemicals. As a result, besides carcinogenic by-products that can be produced by traditional chlorine disinfection, other insidious species can be delivered from the wastewater treatment plants to the water bodies [25,26].

Photocatalytic nanomaterials, if properly supported by polymers, can be successfully applied for wastewater remediation with the advantages of being feasible, cheap and easy to integrate with the already existing water treatment plants [12]. The mechanism of this “green” process is based on the generation of highly-reactive species, namely reactive oxygen species (ROS) (i.e., OH∙, O_2_∙^−^), on the surface of a semiconductor photocatalyst for the mineralization of organic water pollutants and for the death of water pathogens [12]. In brief, when a semiconductor photocatalyst immersed in polluted water is irradiated by photons with energy equal or higher than its band-gap energy, the generated electron–hole pairs can induce the formation of ROS on the photocatalyst surface. The ROS promote oxidation processes, which degrade organic compounds, also refractory, mineralizing them into innocuous species such as CO_2_ and H_2_O, also causing the death of bacteria. The photocatalysis is so promising because the photocatalyst is simply activated by light and can be reused countless times. To date, TiO_2_ and ZnO are among the most studied photocatalysts due to their low cost, biocompatibility, and high photocatalytic efficiency [27,28].

Together with the inorganic photocatalysts, structural ordered polymers can also act as photocatalysts under visible light irradiation [29]. The ease in realizing the desired architectures, their low density, and thermal/chemical durability make them appealing in several applications including the degradation of water pollutants [30]. Among them, covalent organic frameworks (COFs) based on the covalent linkages of selected functional moieties such as imines, boroxines, boronic esters, hydrazones, azines, or ketoenamines, [31] possess high porosity and crystallinity, as well as surface areas comparable to those of porous carbon and metal organic frameworks (MOFs) [32,33,34]. Analogously to the inorganic semiconductors, their photocatalytic properties depend on the formation of electron–hole pairs (band gap of 2.5 ± 0.5 eV), in which the delocalization is due to the π-conjugated ordered systems that provide excellent charge-transfer efficiency. COFs can be obtained in different nanoforms such as nanospheres, nanosheets, and nanotubes [29].

In the case of nanophotocatalysts, the higher reactivity is associated with a high generation of ROS proportional to the amount of the surface-active sites on the photocatalyst immersed in water. Photocatalysts, especially the ones based on TiO_2_ and ZnO, in various shapes and morphologies such as nanoparticles, nanotubes, nanofibers, nanolayers, etc., have been widely studied for the treatment of water contaminated by pollutants including emerging species and bacteria [35,36,37,38,39,40,41,42,43,44,45,46].

In the present review, a selection of photocatalytic polymeric nanocomposites formulated to be active in organic pollutant remediation and disinfection were presented by restricting the temporal search to the last 5 years. In particular, a large part of this review (Section 2 and Section 3) is dedicated to the description of hybrid materials prepared by thermoplastic polymers and TiO_2_ or ZnO employed as photocatalytic nanomaterials. Successively, we will focus on polyporphyrins coupled with graphene so as to exploit the organic photocatalysis under visible light irradiation (Section 4). At the end, we will describe examples of polymeric cryogels, in which the anchorage of ZnO photocatalyst onto the polymeric surface is used for the sponge regeneration after the adsorption of water pollutants (Section 5).

Hybrid systems formulated by using conductive polymers, hydrogels or merely metal NPs as photoactive sites (Au, Ag, Pd) were not reported since they are well described by V. Melinte et al. [47]. Additionally, emerging applications of porous organic polymers (POPs), also including COFs-based materials, in visible-light photocatalysis, were presented in recent reviews and, thus, they are not discussed here [29,31].

It is worth noting that even if the presented materials were tested as photocatalysts for water remediation, they can be employed also in many other application fields such as self-cleaning or self-sterilizing surfaces, air cleaning, or medical applications [48,49].

## 2. Nanocomposites Made of Polymers and TiO_2_ or ZnO Nanostructures

The polymeric nanocomposites described in this section were realized through the combination of TiO_2_ or ZnO nanomaterials with thermoplastic polymers. In particular, TiO_2_ in the forms of nanoparticles or nanotubes, and ZnO in the form of nanoparticles, were employed. Different procedures were described, making it possible to obtain hybrid films in freestanding forms.

### 2.1. Blending of Poly(methyl methacrylate) (PMMA) and TiO_2_ Nanoparticles

**Figure 1:**
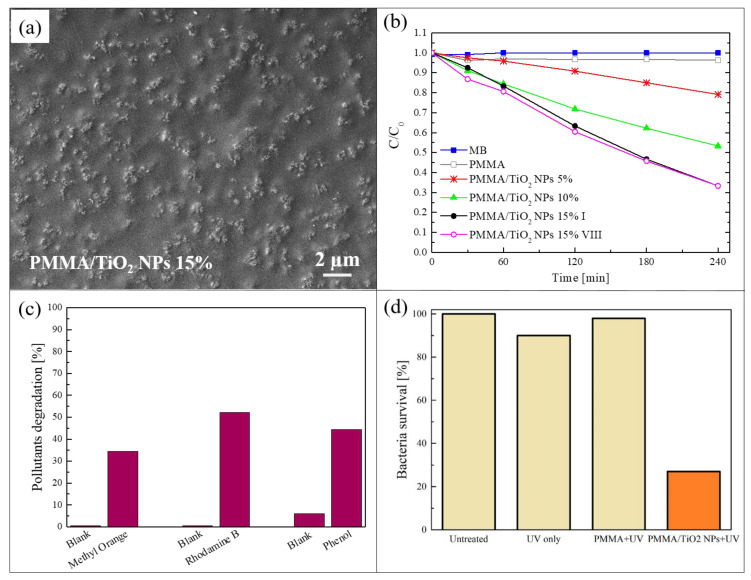
(**a**) Scanning electron microscopy (SEM) image of the back surface of poly (methyl methacrylate) (PMMA)/TiO_2_ sample with 15 wt% of nanoparticles (NPs); (**b**) photocatalytic activity of PMMA/TiO_2_ films evaluated by the discoloration of methylene blu (MB) and stability of the film with 15 wt% of NPs; (**c**) methyl orange (MO), rhodamine B (RhB), and phenol degradation by PMMA/TiO_2_ film with 15 wt% of NPs after 4 h of irradiation; (**d**) antibacterial activity of PMMA/TiO_2_ film with 15 wt% of NPs after 1 h of irradiation [51].

The method of sonication and solution casting [50] was used to realize a blending of poly (methyl methacrylate) (PMMA) and TiO_2_ NPs. This is a simple and low-cost procedure to incorporate photocatalytic nanomaterials in a polymeric matrix. In this process, a polymeric solution and a dispersion of the nanomaterials, added to the same solvent, were sonicated separately and afterwards mixed together and sonicated again. The mixture was then cast into Petri dishes and dried overnight to produce nanocomposite films that were easily peeled off from them. The described procedure made it possible to obtain hybrid films in a freestanding form. The freestanding films produced by the sonication and solution casting method discussed in this section were realized using PMMA as polymeric matrix and commercial TiO_2_ NPs (a mixture of rutile and anatase, with a particle size < 100 nm) as photocatalytic nanomaterials [51]. PMMA was properly selected for its high transparency to visible light as well as superior resistance to the UV radiation. Three different concentrations of TiO_2_ NPs were used: 5 wt%, 10 wt%, and 15 wt%. To study the distribution of the NPs in the polymeric substrate, the nanocomposites were deeply characterized. Figure 1a shows a scanning electron microscope (SEM) image in plan-view of a PMMA/TiO_2_ film with 15 wt% of NPs. This image reports the surface in contact with the Petri dish during the preparation of the sample (named “back surface”). Figure 1a indicates a homogeneous distribution of the NPs on the back surface; the NPs clearly formed small aggregates. Cross-section micrographs of the same composite (not reported here) showed that the TiO_2_ NPs were distributed along the whole thickness of the film, but a higher concentration of NPs was observed in proximity of the back surface [51]. This effect is due to the sedimentation of the NPs during the evaporation of the solvent. The morphology and the distribution of the NPs were shown to be similar for all the sample typologies (i.e., 5 wt%, 10 wt%, and 15 wt% of TiO_2_ NPs) [51].

In Figure 1b, the photocatalytic activity of the three different nanocomposites is reported, exploiting the back surface during the tests. The samples were 1 cm^2^ in size. The photocatalytic aptitude was evaluated through the discoloration of methylene blue (MB) dye, under irradiation with an UV lamp centered at 368 nm and with an irradiance of 2 mW/cm^2^ (irradiance comparable to the UV component of solar spectrum reaching the earth’s surface, in accordance with the International Organization for Standardization (ISO) 10678:2010 test) [52]. The graph shows the variation in the dye concentration as a function of the irradiation time for the samples with the three different amount of NPs. The discoloration of MB in the absence of any samples and the discoloration of a MB solution only in contact with PMMA are reported as references. Before starting with the UV irradiation, the physical adsorption saturation level in the dark was reached for each sample (not reported here), so as to discern between the adsorption phenomena and the photocatalytic ones (this pre-conditioning step was performed for all the photocatalytic tests reported in this manuscript). The photocatalytic activity of these samples increased with the amount of TiO_2_ NPs embedded in the polymeric matrix, as expected. In particular, the samples with 15 wt% of nanomaterials had a discoloration rate of (4.6 ± 0.2) × 10^−3^ min^−1^, and they were able to degrade almost 70% of the dye present in the solution after only 4 h of irradiation. Figure 1b also reports the activity of the films with 15 wt% of TiO_2_ after eight photocatalytic cycles and the efficiency was clearly unchanged. The composites were hence stable and they can be reused many times without any loss of efficiency.

To verify that these materials can be used for the degradation of different organic pollutants, their photocatalytic activity were tested with other two dyes: methyl orange (MO) and rhodamine B (RhB); phenol was also used, so as to exclude any possible dye photobleaching effects [53]. The results for the PMMA/TiO_2_ samples with 15 wt% of NPs are reported in Figure 1c. The specimens were again 1 cm^2^ in size, and were irradiated with the UV lamp described above for a total time of 4 h. The results revealed that the nanocomposites were able to remove ~35% of MO, more than 50% of RhB, and ~45% of phenol present in the solutions. The lower degradation efficiency of MO and phenol compared to MB was probably due to an electrostatic repulsion between MO or phenol and the surface of the sample, due to a negative charge density ascribed to the presence of ester groups. Instead, in the case of RhB a positive charge is present, but only a slight increment in the photodegradation (compared to MO and phenol) was observed because of the presence of many aromatic rings in the molecular structure producing a negative charge density around the RhB molecules.

The antibacterial activity of analogue samples is reported in Figure 1d. *Escherichia coli* (*E. coli*) ATCC25922 was chosen as the model organism, since it is considered an indicator of fecal contamination [54]. The percentages of surviving bacteria after a UV light irradiation for 1 h are reported in Figure 1d. After only 1 h of exposure to light in the presence of PMMA/TiO_2_ sample, the bacteria survival rate was reduced by up to ~30%, in contrast to bacteria exposed to mere PMMA and UV or to UV only, which survive quite well, thus indicating the key-role played by the TiO_2_ NPs.

To demonstrate the versatility of such samples, two different methods were investigated with the purpose of increasing their photocatalytic efficiency. In the first case, the simple boosting of the power lamp was actuated, while for the second case a combination of TiO_2_ NPs with carbon nanotubes (NTs) was realized. The latter is used as a valid strategy to reduce the electron–hole recombination rate, thus raising the photocatalytic properties of the samples [51]. Additionally, the TiO_2_ NPs were functionalized with sensitizers, like porphyrins, so as to extend the light absorption of the composites into the visible region. The as-prepared materials showed, under visible light, the same efficiency obtained under UV light irradiation [51].

### 2.2. Blending of Poly(ethylene terephthalate) (PET) and TiO_2_ Nanoparticles

The research group of A. Lambropoulou has formulated, in recent years, TiO_2_ nanocomposites by using, as a support, bio-plastics such as recycled poly(ethylene terephthalate) (PET), poly L-lactic acid (PLLA), and poly(ethylene furanoate) (PEF); the nanocomposites efficiency was tested on anti-inflammatory/analgesic drugs and also antibiotics in water [55,56,57,58]. Here we report on the formulation of bio-based PET-TiO_2_ photocatalysts and their application for removal of antibiotics in aqueous environment [55]. The hybrid materials were realized by the phase inversion procedure. The procedure involved the dissolution of PET polymer in a solvent or solvent mixture where P25 TiO_2_ was added. Commercial P25 TiO_2_ NPs had a particle size of 20–30 nm and a mixed crystalline structure (~80% anatase and 20% rutile). The obtained viscous solution was then subjected to sonication, so as to disperse the TiO_2_ NPs. The solution was subsequently transferred into glass casting supports, realizing composite films with the desired thickness. The as-prepared films were readily soaked into a non-solvent bath and dried in a vacuum oven, obtaining thin composite films (from 80 to 250 µm) with different photocatalyst content (10 wt%, 30 wt%, and 47 wt% of TiO_2_).

The hybrid polymeric nanocomposites were fully characterized by using different techniques such as X-ray diffraction (XRD), thermogravimetric analysis (TGA), differential scanning calorimetry (DSC) and SEM, whereas their photocatalytic activity was assessed, in relation to a mixture of different pharmaceuticals, under simulated solar irradiation.

**Figure 2:**
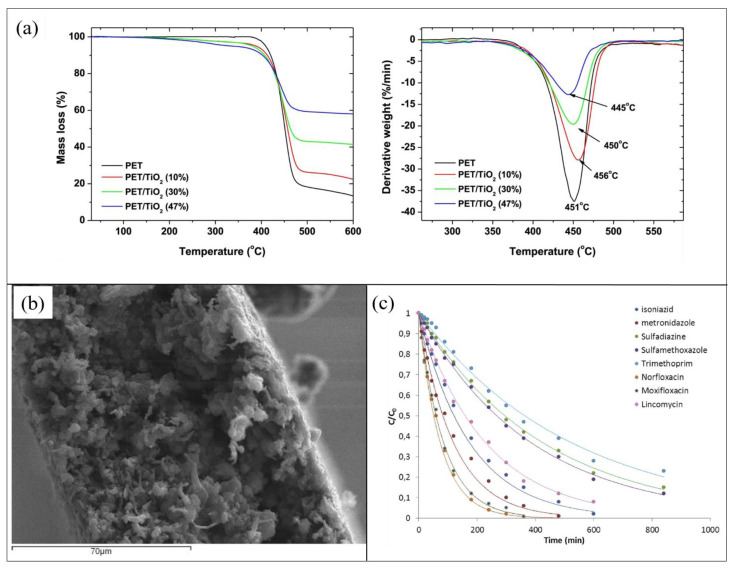
(**a**) Mass loss and derivative weight curves of composite films containing different TiO_2_ contents; (**b**) SEM image of of poly(ethylene terephthalate) (PET) with 10 wt% TiO_2_; (**c**) photocatalytic degradation of antibiotics in the presence of PET 10 %wt TiO_2_ composite films in a wastewater effluent. Figure 2 was adapted from [55], with the permission of Elsevier.

The quantitative determination of photocatalyst incorporated in the PET support was determined by the TGA reported in Figure 2a. The analysis confirmed the final presence of a lower amount of TiO_2_ (about 50% less) compared with the loaded one for all the prepared compositions. This fact was reasonably ascribed to the scarce adhesion of the inorganic filler to the PET matrix that caused a part of its leaching. Additionally, if compared to mere PET sample, adding up to 10% of filler, the thermal stability of hybrid composites increased, whereas higher contents of TiO_2_ determined a depletion degradation temperature, as shown in Figure 2a. Furthermore, XRD analysis indicated that the addition of TiO_2_ NPs into the polymer did not influence their crystallographic form, although the PET crystallinity was affected, as also confirmed from DSC data [55].

Figure 2b shows a SEM image of PET with TiO_2_ 10 wt%, indicating that the prepared composite films were highly porous.

The photocatalytic activity of the synthesized films was investigated for the degradation of a mixture of antibiotic pharmaceuticals: isoniazid, metronizadole, sulfadiazine, sulfamethoxazole, trimethoprim, norfloxacin, moxifloxacin, and lincomycin (1 mg/L for each compound). The photocatalytic degradations of the antibiotic mixture were achieved by a solar simulator equipped with a xenon lamp (1.5 kW of power and 500 W/m^2^ of irradiance). Measurements carried out in the dark, using PET with TiO_2_ 10 wt%, showed that, apart from norfloxacin, which experienced an adsorption of 38%, less than 10% of the other drugs were adsorbed from the modified PET surface. All the PET samples (i.e., with 10 wt%, 30 wt%, and 47 wt% of TiO_2_) efficiently degraded drugs, with higher rates of degradation for higher TiO_2_ amounts. The studied antibiotics were almost completely eliminated after 360 min of solar irradiation. However, when reused for additional purification cycles, only PET with 10 wt% of TiO_2_ maintained good performance for up to five cycles [55]. The efficiency of PET with 10 wt% of TiO_2_ in mitigating water pollution from antibiotics was additionally tested on an effluent obtained from an urban wastewater treatment plant. Figure 2c reports the photocatalytic degradation as a function of the irradiation time. The hybrid material efficiently worked in removing the investigated antibiotics from water, although its kinetic parameters turned out to be lower than those collected from the antibiotic mixture solution prepared in the laboratory [55].

### 2.3. Blending of PMMA and TiO_2_ Nanotubes

**Figure 3:**
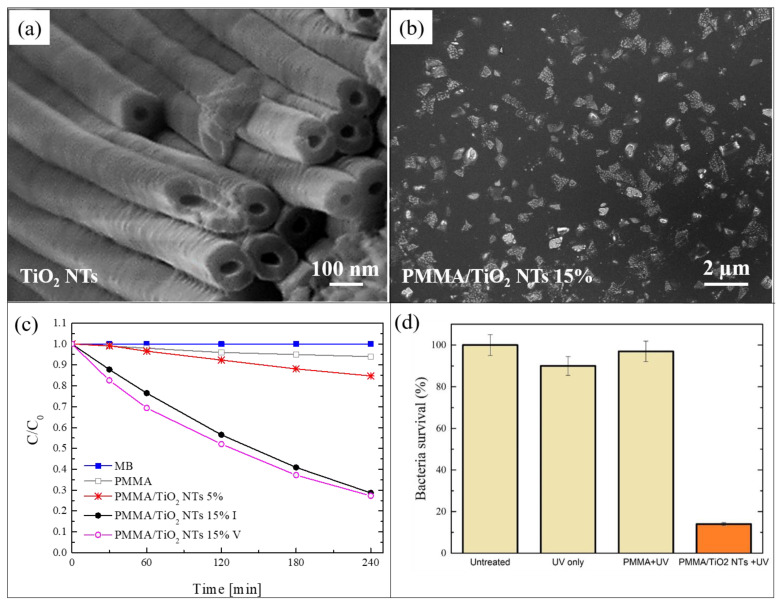
(**a**) SEM image of TiO_2_ nanotubes (NTs) obtained by electrochemical anodization of Ti foils; (**b**) plan-view SEM image of the back surface of the PMMA/TiO_2_ sample with 5 wt% of NTs; (**c**) photocatalytic activity of the PMMA/TiO_2_ NTs films evaluated by the discoloration of MB and stability of the film with 15 wt% of NTs; (**d**) antibacterial activity of the PMMA/TiO_2_ films with 15 wt% of NTs after 1 h of irradiation [59].

In this section, we report a work in which TiO_2_ NTs were mixed with PMMA for the realization of another typology of photocatalytic nanocomposite [59]. The NTs were simply grown by electrochemical anodization of a metallic titanium substrate in electrolytes consisting of ethylene glycol/NH_4_F/H_2_O [60]. Figure 3a shows a SEM image of the obtained nanotubes. After the thermal annealing of the TiO_2_ NTs, the Ti substrates covered by the NTs were immersed in acetone and sonicated for 2 h, so as to induce the separation of the NTs from the Ti foils and their dispersion in the solvent. Such dispersion was then used for the solution casting method in order to realize polymeric nanocomposites using PMMA as matrix. Two different concentrations of TiO_2_ NTs were used: 5 wt%, and 15 wt%. Figure 3b reports a SEM image, in plan-view, of the nanocomposite realized with 5 wt% of NTs. A careful observation of this image indicated that the NTs formed large aggregates, arranged in proximity of the back surface. Additionally, the NTs were aligned along the normal to the surface, as indicated by cross-view SEM images (not shown here) [59].

Despite the observed aggregation, the photocatalytic activity of the realized materials is very satisfying. The photocatalytic performance was tested by irradiating 1 cm^2^ of the back surface of the films with UV light, and measuring the variation in the concentration of the MB solution in which they are immersed. The obtained results, using two films with different contents of NTs, i.e., 5 wt% and 15 wt%, are reported in Figure 3c. After 4 h under UV light, the samples with 5 wt% of NTs were able to degrade almost 20% of the MB, in contrast to the samples with 15 wt%, which degraded more than 70% of the MB. This enhancement in the photo-activity (from 20% to 70%) is due to the higher percentage of TiO_2_ NTs (from 5 wt% to 15 wt%), as confirmed by the constant ratio of the two increments (~3). This significant efficiency (for the samples with 15 wt% of TiO_2_ NTs) is additionally preserved for up to five photocatalytic cycles, as reported in the graph.

The antibacterial activity of these samples is also considerable. Figure 3d reports a survival rate of *E. coli*, after 1 h of UV light irradiation, in the presence of 1 cm^2^ of the film with 15 wt% of NTs; the survival rate was reduced by up to ~15%.

### 2.4. Blending of PMMA and ZnO Nanoparticles

**Figure 4:**
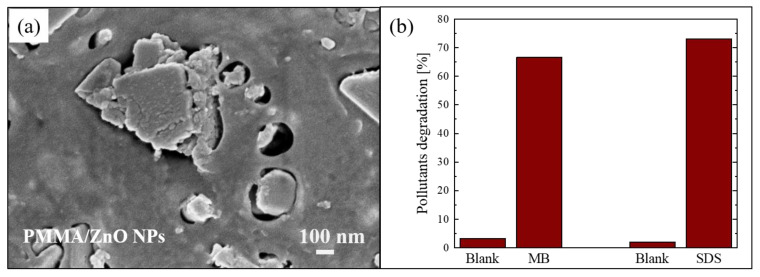
(**a**) Plan-view SEM image of the back surface of PMMA/ZnO NPs sample; (**b**) MB and sodium dodecyl sulfate (SDS) degradation under UV irradiation for 4 h in the presence of PMMA/ZnO NPs sample [61].

Another example of blending of nanostructures with polymer is described by Di Mauro et al. [61]. In this work, the nanocomposite films were again realized using PMMA as a polymeric matrix, but, this time, the PMMA was coupled with ZnO NPs. The ZnO nanoparticles were synthesized using a co-precipitation reaction in alkaline conditions, as reported in the literature [62]. Then, the PMMA/ZnO NPs composites were realized as described in Section 2.1. Figure 4a shows a SEM image in plan-view of a sample with ~4 wt% of ZnO NPs, in which it is possible to observe “holes” in the PMMA matrix filled with the ZnO NPs.

The photocatalytic efficiency of the nanocomposites was tested by the degradation of two organic pollutants: MB dye and sodium dodecyl sulfate (SDS). SDS is an anionic surfactant often used in many cleaning products for its foaming effect, and consequently presents in grey waters. The degradation of these pollutants after 4 h of UV exposure (using a LED lamp centered at 365 nm with an irradiance of 12 mW/cm^2^) in the presence of the photocatalytic nanocomposite (1 cm^2^ in size) is reported in Figure 4b. The results demonstrated that the materials were active in the removal of pollutants; in particular, they degraded about 70% of both tested organic compounds.

## 3. Atomic Layer Deposition (ALD) for the Realization of Nanocomposites Based on ZnO

An innovative approach to realize photocatalytic polymeric nanocomposites involves the use of atomic layer deposition (ALD) to produce thin layers of photocatalysts on polymeric substrates. The ALD is a vapour-based deposition technique in which highly reactive precursors are pulsed alternately into a reactor where a substrate is placed. Each pulse is separated by a purging step with an inert gas. This technique is based on a step-wise and self-limiting gas–solid reaction mechanism and it offers excellent reproducibility, conformality, and uniformity over substrates with different chemical composition, shapes, and dimensions [63]. Traditionally, the ALD on polymers is a challenging process due to the absence of hydroxyl groups on their surface, that are in principle necessary to start the growth, and also due to the thermally fragile nature of the polymers. However, after an extensive investigation in order to find the suitable conditions for the deposition of inorganic photocatalysts on several polymers, it was possible to synthetize hybrid materials with interesting photocatalytic properties. Some examples are reported in this section.

### 3.1. ALD of ZnO on PMMA

**Figure 5:**
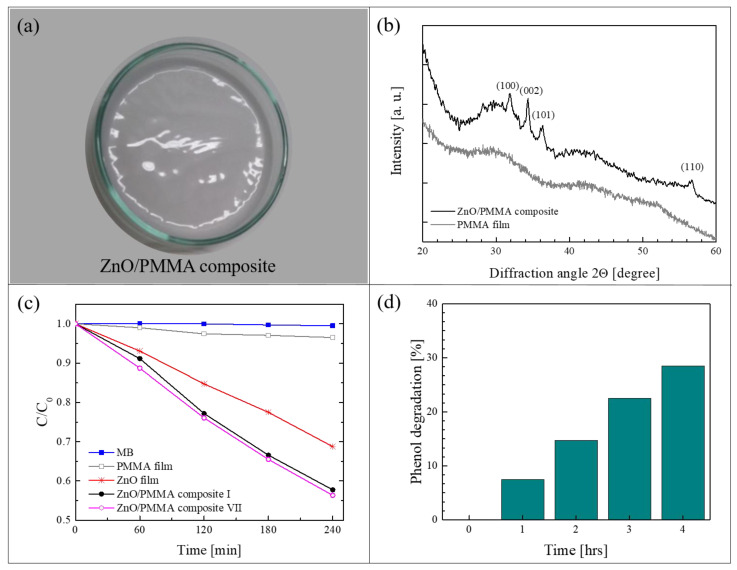
(**a**) Photo of ZnO/PMMA sample in a Petri dish; (**b**) X-ray diffraction (XRD) patterns of PMMA film and of ZnO/PMMA composite film; (**c**) MB photodegradation under UV light in the presence of PMMA film, ZnO/Si film, ZnO/PMMA film, the discoloration of pure MB and the photocatalytic activity of the ZnO/PMMA film after seven cycles are reported too; (**d**) phenol photodegradation under UV light in the presence of ZnO/PMMA film [64].

Figure 5a shows a ZnO/PMMA film realized by the method of solution casting using PMMA powders previously coated with a thin layer of ZnO through ALD [64]. In this work, commercial PMMA powders (molecular weight: 120,000 Da, 0.2–1 mm in diameter) were used as substrates for the deposition of ZnO. The ALD of ZnO was performed using diethyl zinc and de-ionized water as precursors, and N_2_ as carrier and purge gas. The thickness of the ZnO coating was estimated by transmission electron microscopy (TEM) at about 80 nm. After the deposition, the composite films were prepared by dissolving the ZnO/PMMA powders in acetone, casting the solution in Petri dishes and drying the films until the complete evaporation of the solvent. The percentage of ZnO in the composite was estimated, by weighing the PMMA powders before and after the ZnO deposition, to be around 0.14 wt%. Figure 5a is a photo of a ZnO/PMMA sample in a Petri dish. The deposition temperature during the ALD process was maintained at 80 °C, lower than the glass transition temperature of the PMMA. This low temperature was sufficient to obtain crystalline ZnO in the wurtzite structure, as demonstrated by the XRD analysis of a ZnO/PMMA film reported in Figure 5b; the figure also shows, as reference, the XRD pattern of a mere PMMA film, characterized by the typical broad bands related to the amorphous structure of the polymer.

The photocatalytic properties of the polymeric nanocomposites were tested by the degradation of MB dye and phenol under an UV lamp (centered at 368 nm, with an irradiance of ~2 mW/cm^2^). Figure 5c reports the variation in the dye concentration as a function of the irradiation time for the ZnO/PMMA composite film, compared to the activity of a mere PMMA film and of a ZnO film deposited on Si by ALD (with the same thickness of ZnO deposited on PMMA, i.e., ~80 nm). All the samples were 1 cm^2^ in size. The discoloration of pure MB is also reported as control. As expected, no activity was observed without the presence of ZnO (i.e., with MB and PMMA film); the ZnO film on Si was able to degrade 30% of MB, however, the best results were obtained with the ZnO/PMMA composite that was able to degrade more than 40% of MB in the same time frame. In addition, in the same graph, it is worth noting the photo-stability of the ZnO/PMMA composites after seven photocatalytic tests; indeed, the photocatalytic efficiency remained unchanged. The photocatalytic properties of the investigated materials were also tested by the degradation of phenol, a highly toxic compound used in many industrial processes. The results are reported in Figure 5d. The ZnO/PMMA composites were able to remove 30% of the phenol after 4 h of UV light irradiation.

Definitively, this work opens the route for a new strategy for the realization of photocatalytic polymeric composites. Indeed, simply by using polymer pellets covered with the selected photocatalyst, it is possible to realize hybrid materials with different shapes and sizes that are able to efficiently remove pollutants from wastewater.

### 3.2. ALD of ZnO and Ag on PMMA

A further step towards a superior photocatalytic performance is represented by the enrichment of ZnO/PMMA nanocomposites with Ag nanoparticles [65]. Besides the antibacterial properties of Ag [66], the combination of the semiconductor photocatalysts with electron scavengers such as noble metals (e.g., Ag, Au and Pt) increases the separation between the photoexcited electrons and holes, thus reducing their recombination rate, which is one of the main shortcomings of the semiconductor photocatalysts. In detail, noble metals with a Fermi level lower than the conduction band level of the semiconductor create a Schottky junction at the metal–semiconductor interface, so that electrons can be trapped by the metals increasing their separation from the holes [67]. In this work, after the ALD deposition of ZnO films on PMMA flat substrates (2.5 × 2.5 cm^2^ in size), Ag NPs were added on the ZnO surface by plasma-enhanced ALD. The optimization of the Ag deposition on ZnO films at low temperature was obtained due to a meticulous study.

**Figure 6:**
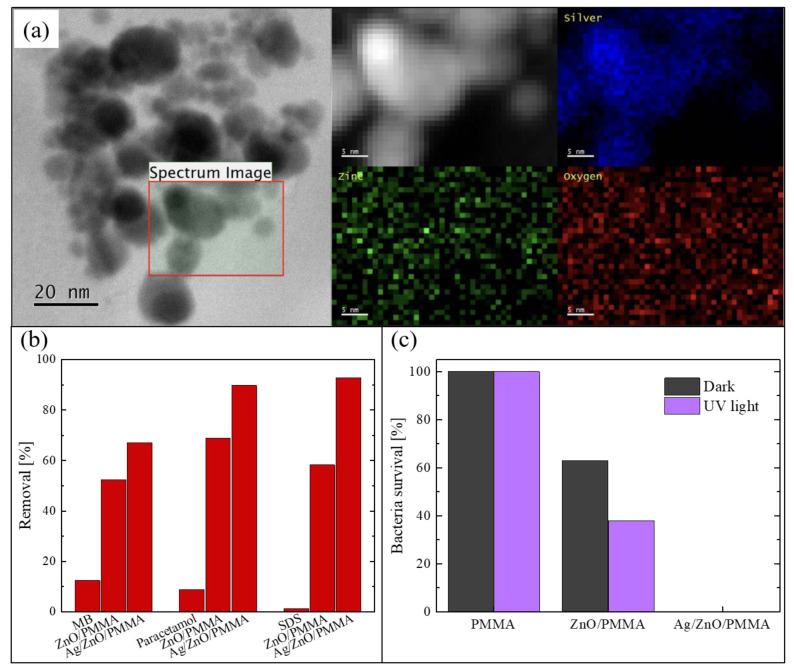
(**a**) Scanning transmission electron microscopy (STEM) image of Ag NPs on the ZnO surface and energy-dispersive X-ray spectroscopy (EDS) signals from silver, zinc, and oxygen; (**b**) degradation of MB, paracetamol, and SDS after 4 h under UV irradiation for blank solutions, in the presence of ZnO/PMMA, and in the presence of Ag/ZnO/PMMA; (**c**) bacterial survival percentage in the dark and when exposed to UV irradiation for 1 h in contact with PMMA, ZnO/PMMA, and Ag/ZnO/PMMA samples [65].

The Ag NPs deposited on ZnO were studied using a probe-corrected scanning transmission electron microscopy (STEM) reported in Figure 6a. Before the analysis, the PMMA substrate was removed by dissolution in acetone after having capped the surface of the sample with a supporting layer of a conductive-epoxy resin. This process was necessary in order to avoid any charge accumulation under the electron beam, due to the insulating nature of the PMMA. The figure shows a plan-view image of the sample, in which it is possible to see the assembled Ag NPs on the ZnO surface. A detailed chemical analysis was performed on the region enclosed in the black square. The corresponding Z-contrast STEM and energy-dispersive X-ray spectroscopy (EDS) spectra were acquired and represented in the 2D map reported in greyscale in the figure. Specifically, the photon counts acquired from silver region are reported in blue, those from the zinc region in green, and those from the oxygen region in red. These analyses confirmed that the black particles correspond to the Ag NPs.

The photocatalytic efficiency under UV light (using a LED UV lamp centered at 365 nm, with an irradiance of 12 mW/cm^2^) was tested by the degradation of three different common organic pollutants: MB, paracetamol, and SDS. In Figure 6b, the percentages of removed pollutants after 4 h of irradiation are reported. The two typologies of composites were tested: Ag/ZnO/PMMA and ZnO/PMMA. The blank solution (i.e., a solution of the pollutant in de-ionized water without the photocatalyst) was always measured for comparison. It is worth noting that the materials enriched with the Ag NPs were always more effective in the degradation of all the three compounds than the composites with only ZnO; in particular, they were able to remove about 90% of both paracetamol and SDS present in the solutions. A lower efficiency is observed in the degradation of MB, presumably due to a different electrostatic interaction with the ZnO positive surface that interacts better with the negative-charged pollutants such as paracetamol and SDS. In any case, this efficiency is better than the efficiency of the samples without Ag NPs.

The antibacterial properties of the Ag/ZnO/PMMA nanocomposites were evaluated by measuring the survival rate of *E. coli* exposed to the samples, both under UV light irradiation and in the dark for 1 h. The obtained data are reported in Figure 6c, and also include the bacteria survival in the presence of mere PMMA and in the presence of ZnO/PMMA. The experiment was run in the dark, and under UV irradiation, in order to estimate the contribution of the photocatalysis. PMMA did not reveal any antibacterial activity neither in the dark nor under UV light, as expected. In the presence of ZnO/PMMA samples, the bacteria survival rate was reduced by up to 60% in the dark and up to 40% under UV light; this difference is due the photocatalytic contribution. Instead, in the presence of Ag/ZnO/PMMA samples, the bacteria survival rate was reduced to 0%, both in the dark and under UV light, thanks to the intrinsic antibacterial properties of Ag NPs.

Ultimately, the addition of Ag NPs to the ZnO/PMMA hybrid materials considerably increased both their photocatalytic and antibacterial activities.

### 3.3. ALD of ZnO on Poly(2,2′-bis(3,4-dicarboxyphenoxy)phenylpropane)-2-phenylendiimide ULTEM^®^ 1000

The growth of inorganic layers onto polymeric substrates is a challenge, as said before (Section 3). In particular, the use of ALD as a synthesis method for the realization of hybrid inorganic/organic materials can involve two different mechanisms: (1) the direct reaction between the ALD precursors and the reactive groups (e.g., -OH and -COOH) of the polymeric surfaces; (2) the diffusion of the precursors into the bulk of the polymers with the successive reaction with the organic polymer—this mechanism is called vapor phase infiltration (VPI) [68]. The occurrence of one of these two mechanisms essentially depends on the nature of the polymeric material. If the selected polymer has a large density of reactive groups on its surface, the involved mechanism during the ALD deposition will be the first one. Instead, the second mechanism will occur with unreactive polymers; in this case, the formation of the inorganic layer will need a higher deposition time and only weak chemical interactions will arise at the interface between the inorganic phase and the organic one. A valid method to convert an unreactive polymer into a reactive one was established. The method consisted in a thermal photo-oxidation process of the polymer [69]. In particular, the selected polymer was the poly(2,2′-bis(3,4-dicarboxyphenoxy)phenylpropane)-2-phenylendiimide ULTEM^®^ 1000 (hereafter simply called ULTEM^®^). This thermoplastic polymer is characterized by high mechanical properties and chemical resistance; however, it is unsuitable for the ALD deposition due to the lack of reactive groups. In order to create -OH and -COOH groups on its surface, the authors submitted 100 µm-thick ULTEM^®^ films to a photo-oxidation process at 60 °C, under UV light exposure at 340 nm, for different time periods: 3, 8, 24, and 48 h [70]. Before the deposition of ZnO by ALD, all the treated samples were characterized to evaluate the creation of reactive functionalities on their surface and this was confirmed both by contact angle measurements and by Fourier transform infrared spectroscopy-attenuated total reflectance (FTIR-ATR) analyses (not reported here) [69]. The wettability of the polymeric material, indeed, increased with the UV exposition and the FTIR signals related to the created functionalities became more evident with the time of exposition. Afterwards, the ULTEM^®^ films were coated with ZnO by ALD. The reactions of the zinc precursor with the reactive species, induced by the photo-oxidation, were assessed by matrix assisted laser desorption ionization time of flight (MALDI-TOF) [69,71]. The samples were characterized by atomic force microscopy (AFM) and compared with the samples in which the ZnO depositions were performed on untreated ULTEM^®^ films. Best results were obtained with the samples photo-oxidized for 8 h, both in terms of ZnO deposition and photocatalytic performance; for this reason, only the results related to these samples are shown here.

**Figure 7:**
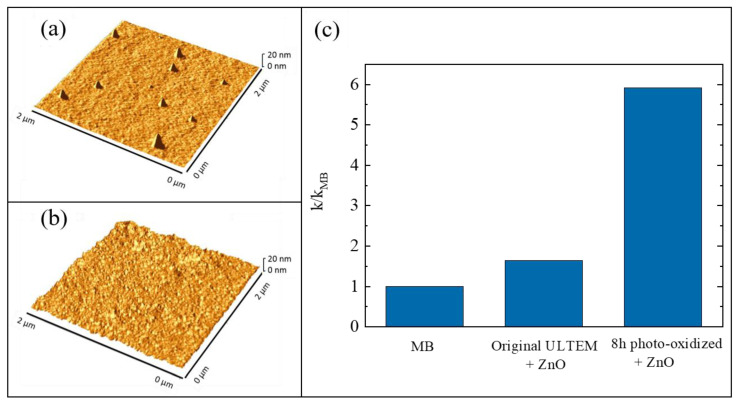
(**a**) Atomic force microscopy (AFM) image of untreated poly(2,2′-bis(3,4-dicarboxyphenoxy)phenylpropane)-2-phenylendiimide ULTEM^®^ 1000 (ULTEM^®^) film covered with ZnO; (**b**) AFM image of 8 h photo-exposed ULTEM^®^ film covered with ZnO; (**c**) photocatalytic efficiency of the two samples after 4 h of UV irradiation [69].

In Figure 7a,b, two 2 µm × 2 µm AFM images are reported; the images are related to a deposition of ZnO on an untreated ULTEM^®^ film and to a deposition of ZnO on a ULTEM^®^ film photo-exposed for 8 h, respectively. A few distinct features standing on the surface, and having an average height of about 18 nm and a reciprocal distance of about 1–2 µm, are observed in Figure 7a; differently, in Figure 7b, the presence of a nano-sized ZnO structure, homogenously distributed on the surface, appeared. These nanostructures were ascribed to the deposited ZnO on the polymeric surface, confirming the necessity to submit the polymer to the photo-oxidation process before the deposition. This was further corroborated by the photocatalytic test under UV light (368 nm with an irradiance of 2 mW/cm^2^), using MB as a target pollutant. Figure 7c shows the values of the photocatalytic reaction rate (*k*) for the untreated ULTEM^®^ covered with ZnO and for the 8 h photo-exposed ULTEM^®^ after ZnO deposition, both normalized to the *k* value found in the absence of any photocatalyst (*k_MB_*). According to the Langmuir–Hinshelwood model, the reported photocatalytic reaction rates were calculated by the following reaction: *ln(C/C_0_) = −kt*, where *C* is the concentration of MB, *C_0_* is its starting concentration and *t* is the irradiation time [12].

This work demonstrates how, after an ad hoc modification of the polymeric surface, the ALD can be used for the realization of hybrid materials that are effective as photocatalysts.

## 4. Graphene/Polyporphyrins Hybrid Materials

The realization of graphene-based hybrid materials for photocatalytic applications has received a lot of attention in recent years due to the extraordinary properties of graphene [72,73]. The combination of graphene-based materials with photocatalysts allows the realization of hybrid systems with improved photocatalytic performances for both organic and inorganic semiconductors. The role of the graphene is to promote the separation as well as the transfer of photo-generated charges. However, some drawbacks arise from the intrinsic “shielding effect” and the radical scavenger activity of graphene platforms, especially in the cases of graphene oxide and reduced graphene oxide [73,74]. These negative effects impose the use of a limited quantity of graphene oxide (lower than 5%) when used as co-photocatalyst, also determining a decrease in electron–hole separation efficiency [74].

Another interesting aspect extensively studied for the photocatalysis topic concerns the realization of photocatalytic systems active under visible light. For this purpose, the use of photosensitizers, such as porphyrins, has received great consideration [18,51,75]. These organic molecules have a great potential as visible light photocatalysts. However, as reported in the literature, their chemical structures, and the morphology and size of their self-assembly aggregates, can negatively impact on their photocatalytic efficiency. These factors can indeed promote the fast recombination of photo-generated charges [76]. The coating of high-quality graphene with porphyrins can be a valid solution in order to overcome the above mentioned limitations. The hybrid materials presented in this section try to efficiently reach this goal.

### 4.1. 3D Graphene Supported by Nickel Foam Coupled with Porphyrin-Based Polymers

An interesting example of hybrid materials that combine the properties of graphene with the properties of porphyrins is represented by the work of Ussia et al. [77]. Freestanding photocatalytic materials, active under visible light, were realized through 3D graphene supported by nickel foam and decorated by polyporphyrins. The first step of the synthesis procedure was represented by the chemical vapour deposition (CVD) of graphene on nickel foam pieces, these latter used as 3D scaffold templates. Separately, by simple polymerization processes, two types of cyclic polyporphyrins, homo-polyporphyrin (homo-PPr) and co-polyporphyrin (co-PPr), were synthetized (the details of the polymerization procedures are reported in [77]). The use of porphyrin in a cyclic polymeric form instead of simple porphyrin molecules was chosen to prevent the formation of inactive porphyrin aggregates that, as said before, negatively influence the photocatalytic performance. After the polymerization, the nickel foam covered by graphene (GF) was used as solid support and embedded with the polyporphyrins in order to realize the freestanding device. The procedure was very simple: the opportune amount of each polyporphyrin was dissolved in dimethylformamide, then the GF was immersed in the obtained solution overnight; afterwards, the GF was removed from the solution and it was dried under vacuum at 50 °C.

**Figure 8:**
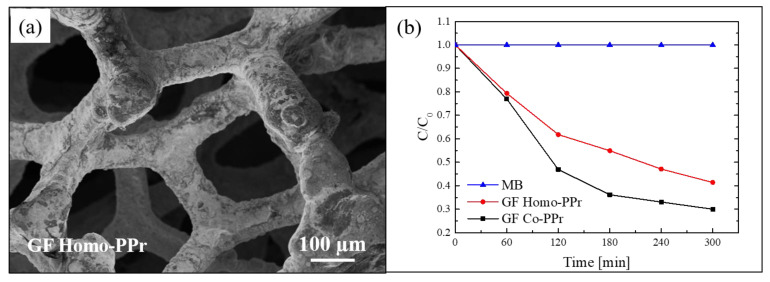
(**a**) SEM image of graphene (GF) homo-polyporphyrin (homo-PPr); (**b**) photocatalytic activity GF co-polyporphyrin (co-PPr) and GF homo-PPr compared to the discoloration of pure MB under visible light irradiation [77].

SEM analyses were performed to observe the morphology of the hybrid materials. In Figure 8a, a SEM image of a sample composed by homo-PPr on GF is reported. The image indicated a total coating of the foam by the polymer. The SEM images of the samples with the co-PPr revealed a similar morphology. This simple assembly procedure offers the possibility to realize systems in which a direct contact between the graphene and the photosensitizer is guaranteed by non-covalent π–π interactions at the interface. At the same time, the polymeric coating protects the graphene by the direct exposure to light and radicals, reducing in this way the “shielding effect” and the radical scavenger phenomena; thus, by optimizing the charge transfer to graphene, the electron–hole recombination rate is reduced.

The photocatalytic activity of these systems was tested by the degradation of MB under visible light using a xenon lamp operating at 1.5 mW/cm^2^ and equipped with a cu-off filter (λ > 400 nm). The obtained results, after reaching the adsorption–desorption equilibrium, are reported in Figure 8b. Both systems (i.e., co-PPr on GF and homo-PPr on GF) showed a considerable photocatalytic efficiency. In particular, the best performance of the system with co-PPr, if compared with the system with the homo-PPr, was ascribed to the presence of isolated porphyrins along the cyclic chains that better prevent the agglomeration.

### 4.2. Nickel-Free Graphene Foam Coupled with Porphyrin-Based Polymers

**Figure 9:**
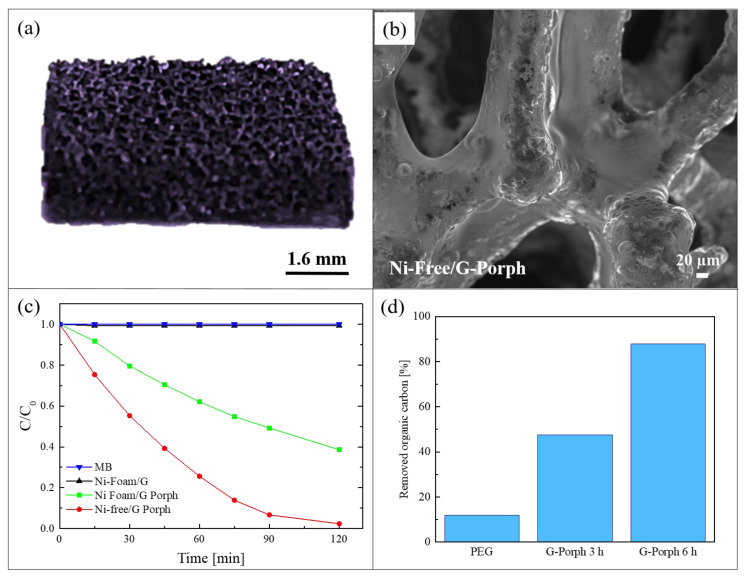
(**a**) Photograph of Ni-Free/G-Porph sample; (**b**) SEM image of Ni-Free/G-Porph sample; (**c**) photocatalytic activity of Ni-Foam/G, Ni-Foam/G-Porph, Ni-Free/G-Porph toward the degradation of MB; (**d**) Total organic carbon (TOC) measurements of pristine PEG after 6 h of irradiation, and in the presence of Ni-Free/G-Porph after 3 and 6 h of irradiation, respectively [78].

As demonstrated by the previous work, the combination of graphene-based materials and porphyrin polymers offers efficient photocatalytic systems active under visible light. However, for a real water application, the previous reported hybrid materials present a serious issue due to the presence of nickel. This metal is indeed a common allergen, harmful for aquatic and human life. In order to overcome this problem, Ussia et al., in a successive work, realized a hybrid material nickel-free based on graphene and polymeric porphyrin [78]. The main difficulty of the work lied in the removal of the nickel substrate by etching it in an acid environment while avoiding the 3D graphene structure collapsing. Many papers in the literature reported that the etching procedure in HCl to eliminate the nickel substrate requires the protection of graphene with a polymeric layer of commercial PMMA or polydimethylsiloxane [79]. However, when the protecting polymer is removed from the graphene surface by dissolution in a solvent, the rapid collapse of 3D graphene is observed. Consequently, a common strategy to avoid the 3D collapse consists in the non-removal of the protective polymer coating. Considering this strategy, in the present work, the protective layer was realized with the polymeric porphyrin, exploiting in this way its double role as the protective layer and the photocatalytic material. Briefly, after the CVD deposition of graphene on nickel foam, a polymeric porphyrin ring was spin-coated on the graphene surface (these samples were named Ni-Foam/G-Porph). When the nickel substrate was etched in HCl (these samples were hereafter called Ni-Free/G-Porph), the 3D structure did not collapse, as shown in the Figure 9a. The preservation of the 3D porous morphology of the Ni-free hybrid materials was confirmed by SEM analysis, as reported in Figure 9b.

The photocatalytic efficiency of 1 cm^2^ of the obtained materials was tested under a solar simulator with a light irradiance of 100 mW/cm^2^ and using the following pollutants: MB dye, the water soluble polyethylene-glycol (PEG) used in many personal care products, and the pesticide 2,4-dichlorophenoxyacetic acid. For the sake of synthesis, here we summarized the results obtained with the first two pollutants. In Figure 9c, we report the MB reduction as a function of irradiation time. The photocatalytic tests were performed after the adsorption/desorption equilibrium was reached for all the studied samples. The Ni-Foam/G samples did not reveal any photocatalytic activity, as expected given the lack of porphyrins. The best photocatalytic performance was exhibited by the Ni-Free/G-Porph samples, which degraded almost 95% of MB in only 120 min of irradiation, while the sample with nickel (i.e., Ni-Foam/G-Porph) was able to degrade only 60% of MB in the same time frame. These results revealed that, in the case of the Ni free samples, the electron-transfer between porphyrin and graphene is more effective. The capacity of the Ni-Free/G-Porph samples in mineralizing the PEG is reported in Figure 9d. The measures were performed by a total organic carbon (TOC) analyzer with up to 6 h of irradiation. The Ni-Free/G-Porph samples were able to mineralize more than 85% of PEG after 6 h of solar light irradiation, thus demonstrating the effectiveness of the photocatalytic process. In the graph, the photodegradation of pristine PEG is also reported for comparison.

In the paper, the free radical and hole scavenging tests were also performed in order to determine the individual contribution of the ROS on the photocatalytic activity of the samples. The tests revealed that the predominant species involved in the photodegradation of contaminants is the singlet oxygen [78].

## 5. Hybrid Nanosponges

The photocatalytic process can be also integrated as an effective method for the removal of contaminants on adsorbent materials. In fact, one of the most feasible, efficient, and low-cost methods for water remediation is based on adsorption. Many adsorbent materials have been developed to date: carbon-based materials, polymers, organic–inorganic composites, etc. [80]. However, despite the high efficiency of all these materials in removing pollutants from water, major limitations include their regeneration as well as the final disposal of contaminants. The hybrid materials presented in this section overcome this problem by combining photocatalysts, with remarkable photocatalytic aptitude, and polymers, with extraordinary adsorption ability.

### ALD of ZnO on Poly(2-hydroxyethylmethacrylate) (pHEMA)

**Figure 10:**
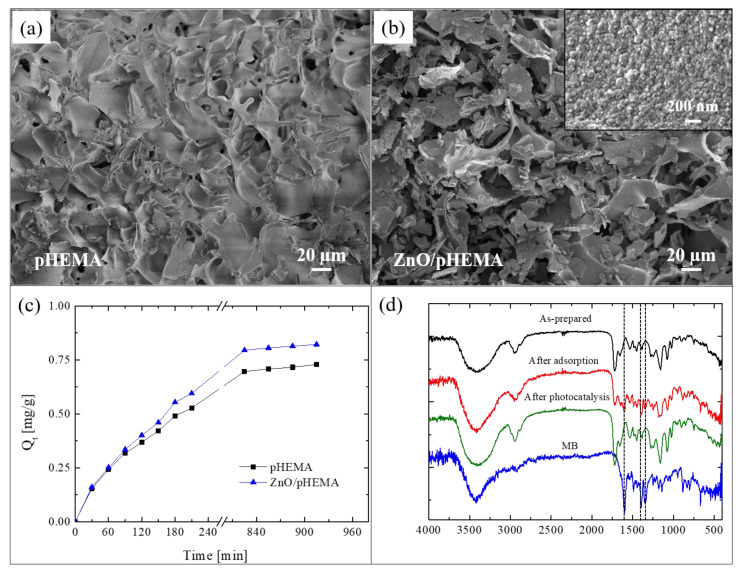
(**a**) SEM image of poly(2-hydroxyethylmethacrylate) (pHEMA) sample; (**b**) SME image of ZnO/pHEMA sample; (**c**) MB adsorption capacity versus time for pHEMA and ZnO/pHEMA samples; (**d**) Fourier transform infrared spectroscopy (FTIR) spectra measured on Zn/pHEMA samples: as-prepared, after MB adsorption, and after UV light irradiation; the spectrum of pure MB is also reported as reference [81].

Here, we present an innovative approach for the realization of reusable photocatalytic sponge based on ZnO and poly(2-hydroxyethylmethacrylate) (pHEMA) [81]. The super-adsorbent polymeric material was synthesized via the green method of cryopolymerization in water, using 2-hydroxyethylmethacrylate (HEMA) as monomer, grapheneoxide as filler, and performing the polymerization at −16 °C. After that, nanolayers of ZnO were deposited by ALD at 80 °C on pHEMA cryogel, taking advantage of the hydroxyl pendant groups of pHEMA that covalently link the polymer with the ZnO. The as-realized freestanding photocatalytic sponges were submitted to a deep characterization [81]. Figure 10a reports a SEM image of pHEMA before the ZnO deposition; the image revealed a jagged microporous surface, with a random 3D network. The SEM image of a pHEMA sample after the ZnO deposition is shown in Figure 10b; it did not reveal any substantial differences with the previous one (Figure 10a), thus demonstrating the high conformability of the ALD technique. In the inset of Figure 10b is reported a high magnification of the ZnO/pHEMA surface; the image clearly shows the typical morphology of ZnO, which is composed of small grains uniformly distributed on the polymeric surface [20].

To evaluate the adsorption abilities of the studied sponges, both samples (i.e., pHEMA and ZnO/pHEMA) were immersed in a MB solution until the adsorption equilibrium was reached. The results are shown in Figure 10c. At time *t*, the adsorption capacity *Q_t_* (mg g^−1^), reported in the graph, was calculated by the following equation [82]:

$$Q_{t}=\frac{\left(C_{0}-\;C_{t}\right)V}{W}$$

where *C_0_* (mg L^−1^) is the starting concentration of MB, *C_t_* (mg L^−1^) is the concentration of MB at time *t*, *V* (L) is the volume of the solution, and *W* (g) is the amount of the used adsorbent material. The adsorption capacity at the equilibrium was 0.73 ± 0.01 mg g^−1^ and 0.82 ± 0.02 mg g^−1^ for pHEMA (without ZnO) and ZnO/pHEMA, respectively. So, in the presence of ZnO, the adsorption capacity of pHEMA is slightly increased. The regeneration of the materials, exploiting the presence of ZnO nanolayers, were evaluated by three adsorption tests with MB followed by photocatalytic processes (performed under UV lamp, centered at 368 nm, with an irradiance of 4 mW/cm^2^, for a total time of 5 h). After the second and third cycles, the pHEMA sample exhibited a sensible decrease in the adsorption ability; on the contrary, the sample covered with ZnO showed a comparable adsorption efficiency during the three cycles, thus demonstrating that the exposure to the UV lamp was effective in regenerating the material thanks to the photocatalytic process (for more details, see [81]).

To support the effectiveness of the photocatalytic regeneration, FTIR analyses were performed on ZnO/pHEMA samples. Figure 10d reports the spectra of an as-prepared sample, after an adsorption cycle, and after the photocatalytic regeneration. The spectrum of pure MB was reported as reference. The as-prepared sample did not show the typical peaks of MB at 1600 cm^−1^, 1354 cm^−1^, and 1340 cm^−1^, as expected; these peaks instead appeared after the adsorption process, but they were not more visible after the photocatalytic process. The observed differences between the spectra before and after the photocatalysis were not observed for the samples of pHEMA, thus demonstrating the key role of ZnO.

Cryogel formulation provides additional benefits if compared to its bulk form. Indeed, the interconnected macroporous architecture offers water fast-diffusion channels, causing an in-depth access to the photoactive sites, thus enhancing the photocatalysis. Additionally, lower back pressure without pore obstruction is manifested when filtering systems are used for heterogeneous solutions [83].

These results reveal an innovative approach to solve many of the problems associated with the use of adsorbent materials for water treatment, opening the route for a large application in this field.

## 6. Conclusions

This paper aimed to provide an overview on different types of hybrid nanocomposites made of polymers and semiconductors for applications in photocatalysis. The properties of nanostructured semiconductor photocatalysts (TiO_2_ and ZnO in the forms of nanoparticles, nanotubes, and nanolayers) were combined with the properties of different polymeric matrices. In addition, 3D graphene coupled with porphyrin-based polymers were presented as efficient photocatalytic materials under visible light irradiation. In this case, the photocatalytic activity is due to the polyporphyrins, while the graphene plays a key role in increasing the photocatalytic efficiency by reducing the electron–hole recombination phenomena.

Several methods to realize hybrid inorganic-organic materials were illustrated, and all of them are easily up-scalable from laboratory to process scale.

The remarkable photocatalytic activity of the nanocomposites was proved by the degradation of organic pollutants in aqueous solution: dyes, phenol, pesticides, drugs, and personal care products. The antibacterial properties of the samples were tested using *E. coli* as a model organism. Even though the hybrid materials were specifically tested for the degradation of water pollutants, they could find applications as efficient photocatalysts in other fields such as self-cleaning, air cleaning, and medical applications.

This review, covering many aspects of new hybrid nanomaterials based on polymers and both organic and inorganic semiconductors, including their photocatalytic aptitude, could be of help for scientists and students interested and involved in these domains.
